#  Trabalhadores(as) em plataformas digitais: precarização e sobrevivência 

**DOI:** 10.1590/0102-311XPT000624

**Published:** 2024-03-11

**Authors:** Sergio Roberto de Lucca

**Affiliations:** 1 Faculdade de Ciências Médicas, Universidade Estadual de Campinas, Campinas, Brasil.

Nos anos 1980, o acelerado desenvolvimento das tecnologias de informação e comunicação (TIC) e o processo de financeirização da economia global inaugurou uma nova fase do capitalismo, da economia digital e de exploração da força de trabalho global, ao modular os processos tecnossociais em um modelo mediado pelas tecnologias e plataformas digitais.

Antes mesmo das plataformas, as tecnologias digitais já existiam para gerir e controlar o trabalho. Como exemplos, Antunes & Braga ^1^ citam os “infoproletários” para designar a exploração e controle virtual dos(as) trabalhadores(as) de *call center* e dos(as) programadores(as) de software, que vivenciam uma autonomia paradoxal, cada vez mais reféns da produtividade de quem os contrata.

Na divisão internacional do trabalho, as tecnologias digitais, além de conectar e articular várias formas produtivas - da indústria aos serviços -, também conseguiu obter maior eficácia produtiva, à medida que intensificou o trabalho e a vigilância sobre os(as) trabalhadores(as). Além disso, em um cenário de desemprego estrutural e de trabalho informal, mais comuns nos países periféricos do Sul Global, a *gig economy*, ou economia de bicos, aparece como única saída de sobrevivência para 61% dos trabalhadores(as) informais no mundo, já que o desemprego atinge cerca de 207 milhões de pessoas ^2^.

No Brasil, a informalidade também é elevada e dados recentes do Instituto Brasileiro de Geografia e Estatística (IBGE) apontam que 41% da população ocupada trabalhavam sem carteira ou por conta própria ^3^. Em 2022, cerca de 1,5 milhão de brasileiros(as) realizavam trabalho por meio de plataformas digitais de serviços, enquanto 628 mil utilizavam plataformas de comércio. Nesse período, entre os trabalhadores autônomos, 9,8% da população ocupada realizava trabalho remoto e 7,7% teletrabalho exclusivamente ^4^. O enorme contingente de trabalhadores(as) sem proteção social e com fatores de risco potenciais é um desafio para a saúde pública.

Nesse cenário, no livro *Trabalho por Plataformas Digitais: Do Aprofundamento da Precarização à Busca por Alternativas Democráticas*^5^, os autores Rafael Grohmann & Julice Salvagni problematizam o impacto das mudanças promovidas pelas plataformas digitais como um novo tipo de organização de trabalho que se reapropria da informalidade para extração do mais valor, além de aumentar a precariedade e a exploração de trabalhadores(as), em um cenário sem precedentes de trabalho sob demanda, mediadas por empresas que se utilizam das tecnologias e plataformas digitais como forma de exploração da força de trabalho.

No capítulo 1, sobre o trabalho por plataformas digitais, os autores nos alertam sobre as diferenças conceituais entre plataformização e uberização e elegem como campo de investigação todo o trabalho que é mediado, organizado, controlado e/ou governado pelas plataformas. Pontuam que o trabalho continua sendo uma atividade humana que vai além das plataformas digitais; entretanto, o amplo processo de informatização modificou as formas de gerenciamento, controle e organização do trabalho, com ênfase no autogerenciamento subordinado e de desempenho individual, em que a subjunção do trabalho informal pelo capital transformou cada trabalhador e trabalhadora em “trabalhador sob demanda”. Ou seja, apesar de a circulação de pessoas e mercadorias se dar por intermédio das plataformas, ainda existe uma dependência de várias formas de trabalho vivo e com vigilância algorítmica dos seus rastros digitais.

No capítulo 2, sobre quem são os trabalhadores por plataformas, os autores destacam que, no neoliberalismo, o uso das tecnologias, a serviço do capital, não é neutro e sua dispersão espacial é, ao mesmo tempo, global e local em função do setor econômico, das habilidades e complexidades do tipo de trabalho requerido (de microtarefas repetitivas à desenvolvimento de softwares) e das ocupações (entregadores, motoristas, professores, empregados domésticos, freelancers etc.). Entrecortados por marcadores sociais de desigualdades e diferenças, no trabalho por plataformas se entrelaçam clivagens de raça, gênero, território, além de, em seus contextos, culturas e dinâmicas interseccionais. Embora os entregadores e motoristas de aplicativos sejam mais conhecidos, eles representam a ponta do iceberg. No universo da precarização do trabalho digital, estão incluídos os desenvolvedores de inteligência artificial (IA), fazendeiros de cliques, criadores de conteúdo e freelancers, totalizando mais de 160 milhões de trabalhadores no mundo, a maioria do Sul Global. Somente no Brasil, há mais de cinquenta plataformas de microtrabalho e microtarefas de baixa complexidade e elevada repetitividade.

No capítulo 3, sobre os mecanismos do trabalho por plataformas, a arquitetura assentada sobre o gerenciamento algorítmico e a datificação representa as principais alavancas na financeirização e nos ganhos de capital, materializados pela racionalidade neoliberal. O gerenciamento algorítmico do trabalho é um conjunto de práticas de supervisão, governança e controle sobre os(as) trabalhadores(as) de forma remota. Além disso, no design original, as plataformas desconsideram as diferenças de raça e gênero e são projetadas para desarticular a comunicação e organização entre esses(as) trabalhadores(as).

No capítulo 4, sobre saídas para o trabalho de plataformas, os autores apontam algumas brechas e fissuras na lógica das plataformas digitais para driblar o algoritmo e resistir às pressões individuais cotidianas. Entretanto, no âmbito coletivo, é necessário que a maioria se reconheça como força de trabalho explorada, não como empreendedores. Embora as pautas entre os trabalhadores de aplicativos e plataformas não sejam homogêneas, considerando-se as leis de trabalho digno e decente da Organização Internacional do Trabalho (OIT), são destacados cinco princípios para empregos em plataformas digitais, seja nas ruas ou em casa: remuneração, condições de trabalho, contratos, gestão e representação. No Brasil, em 2023, a Aliança Nacional dos Entregadores de Aplicativos reivindicou, sobretudo, a formalização das relações de trabalho, da proteção social para acidentes de trabalho, das garantias de remuneração, da liberdade de associação e da sindicalização. Sobre a rigidez das plataformas, algumas universidades, em parceria com laboratórios de inovação, estão desenvolvendo um sistema digital público, para o livre acesso dos trabalhadores. Uma alternativa é o cooperativismo de plataforma.

No capítulo 5, sobre o cooperativismo de plataforma, os autores discutem como essas tecnologias podem ser apropriadas pelos trabalhadores, a partir de um movimento de luta de classes, ou seja, de baixo para cima, incluindo a possibilidade de políticas públicas, considerando sete princípios internacionais das cooperativas de trabalho: adesão livre e voluntária; gestão democrática; participação econômica dos membros; autonomia e independência; educação, formação e informação; intercooperação; interesse pela comunidade. As iniciativas e experiências internacionais no cooperativismo de plataformas são relevantes e podem servir de referência; entretanto, os autores pontuam as dificuldades em “tropicalizar” modelos do Norte Global e defendem a adaptação do cooperativismo de projetos à cultura e realidade brasileira, tanto em âmbito regional quanto nacional.

E qual é o futuro do trabalho por plataformas? Para os autores, a resposta depende da capacidade de organização dos movimentos sociais, considerando-se as brechas e fissuras em torno das formas emergentes de solidariedade e estruturação.

O desenvolvimento das tecnologias digitais modificou os processos e a organização do trabalho e amplificou globalmente as relações de trabalho individualizadas e invisibilizadas. A apropriação hegemônica das TIC pelo capital, embora não substitua o trabalho humano, visto que as máquinas não criam valor, potencializa e intensifica o trabalho, o controle e a vigilância sobre os assalariados formais (nos setores da indústria 4.0, dos serviços e do comércio) e informais, incluindo a enorme força de trabalho tipificada nesse livro como parte de um iceberg sem saída de “escravidão digital” ^6^.

A pandemia de COVID-19 normalizou e ampliou o trabalho digital, que abrange as atividades em *home office* e nas ruas, com entregadores e motoristas por aplicativos, também intensificou a precarização do trabalho e eliminou a separação entre a vida privada e o trabalho. Nesse cenário, é perceptível o aumento do sofrimento e dos transtornos mentais, para aqueles que trabalham em casa, e dos acidentes graves, entre os motofretistas ^7^. O desafio do Sistema Único de Saúde (SUS) e sua plataforma digital (e-SUS) é conseguir adequar sua rede para acolher as demandas dessa força de trabalho cada vez mais refém das tecnologias digitais. A agenda da telessaúde pode ser o caminho mais promissor.
